# The Report of Three Rare Cases of the Niemann-pick Disease in Birjand, South Khorasan, Eastern Iran

**Published:** 2017

**Authors:** Samaneh NOROOZI ASL, Rahim VAKILI, Nosrat GHAEMI, Peyman ESHRAGHI

**Affiliations:** 1 Pediatrics Endocrinology Department, Mashhad University of Medical Sciences, Mashhad, Iran; 2 Endocrinology Department, Mashhad University of Medical Sciences, Mashhad, Iran

**Keywords:** Niemann–Pick disease type C (NP-C), Splenomegaly, Infants, Supranuclear gaze palsy

## Abstract

Niemann–Pick disease type C (NP-C) is a rare neurovisceral and irreversible disease leading to premature death and disabling neurological signs. This autosomal recessive disease with incidence rate of 1:120000 is caused by mutations in either the NPC1 or the NPC2 gene, which leads to accumulation of cholesterol in body tissues especially brain and progressive neurological symptoms. NP-C is characterized by nonspecific visceral, neurological and psychiatric manifestations in infants. The neurological involvement is typically proceeded by systemic signs (cholestatic jaundice in the neonatal period or isolated spleno-or hepatosplenomegaly in infancy or childhood).

Early detection of NPC is important so that therapy with miglustat can delay onset of neurological symptoms and prolong survival. We describe here three infants from Birjand, South Khorasan, eastern Iran in 2016 with splenomegaly and different neurological signs that diagnosis was confirmed by genetic study. In all of them, NPC-509 was pathologically increased. They also had an unreported homozygous mutation (c.1415T>C, p.Leu472Pro) in exon 9 of the NPC1 gene. We found unreported homozygous mutation in NPC gene.

Knowing this mutation is significant to our people. Genotype-phenotype correlations for this specific mutation needs to be further studied.

## Introduction

Niemann-Pick disease type C (NP-C) is a rare, progressive genetic lysosomal lipid storage disease caused by mutations in the NPC1 or NPC2 gene and incidence rate of 1/120000. It is a highly heterogeneous disease, characterized by visceral, neurological and psychiatric manifestations that can be presented alone, or in specific or non-specific combinations (1). Moreover, age at onset and disease course is different. Very early-onset patients are often diagnosed based on isolated systemic manifestations, but patients most often present during childhood with one or more neurological manifestations such as abnormal saccadic eye movement disorders, cerebellar ataxia, learning problems, gelastic cataplexy and clumsiness (2).

Early diagnosis is essential so that therapy with miglustat can be initiated as soon as neurological symptoms appear in order to slow the progression of neurological damage.

## Case Report

The first case is a 3.75-yr old boy, referred to the Imam Reza Hospital, Pediatric Endocrinology and Metabolic Service, Mashhad, Iran in 2016. The patient is the first offspring of a first-cousin marriage from Birjand, South Khorasan, Iran, with birth weight of 2250 gr, 47 cm height, and head circumference of 34 cm. The patient had a history of neonatal jaundice from 40 days earlier. 

Developmental milestones were delayed, the head holding was at five months, at nine months he was able to sit independently, and at the age of two years, he started to walk on feet. At six months, the patient was admitted to hospital due to gastroenteritis, and abdominal examination demonstrated splenomegaly. Cell blood count and liver transaminase function tests were normal and peripheral blood smear had no pathological finding. 

In fundoscopy, cherry red spot was not detected. For further investigation, bone marrow aspiration was performed, which exhibited foamy cells. Thereafter, enzyme assay was carried out, reporting normal activity of glucuronidase, galactokinase, and sphingomyelinase. There was no problem until 2.5 yr of age when he went to kindergarten. Speech and learning were acceptable; however, from the age of three, he started to suffer from ataxia and frequent falling, impaired speech, and dysphagia. At referral, spasticity, cerebellar dysfunction, dystonia, and splenomegaly were noted. 

At the age of two, enzyme assay was re-performed, which was normal, but with probable diagnosis of Gaucher’s disease, enzyme replacement therapy was carried out for eight months. However, developmental regression trend was accelerated in the recent months and currently, he is not able to walk, sit, or swallow.

He has no history of seizure or hearing impairment.

Electroencephalogram revealed generalized sharp waves and in magnetic resonance imaging, sulcal widening and frontoparietal furrows were reported. Eye examination was abnormal and vertical supranuclear gaze palsy was detected. Moreover, in ultrasonography, spleen size was 41*122 with normal echo pattern and without hepatomegaly. Considering the combination of systemic and neurological symptoms and risk prediction score of higher than 70, NPC was suspected, accordingly, genetic analysis was performed and the diagnosis confirmed. 

(Order no: 62262135, Centogene Company) The second case is a three-yr-old girl, from Birjand, South Khorasan, and Iran. This patient is the cousin of the first case. She is the second child of non-related parents. The older sibling is healthy. The patient had birth weight of 2400 gr, height of 49 cm, and head circumference of 34 cm. By the age of nine months, she was developmentally normal and could sit and crawl. At the age of seven months, she was examined due to irritability and anemia pulse splenomegaly was diagnosed (ultrasonography showed spleen size of 108 mm and normal echo). Since the age of nine months, she developmentally regressed and never obtained the capability of independent walking and talking. 

Liver function tests were normal and mild hypochromic microcytic anemia was detected in peripheral smear. 

The high-performance liquid chromatography of serum amino acids and MS/MS examinations, and enzyme assay for lipid storage disease was normal and in BMA, foamy cells were noted. In neurological examination, spasticity, platter dystopia, dysphagia, and clumsiness were observed. The patient also had history of frequent focal and generalized seizures controlled by antiepileptic drugs. In abdominal examination, splenomegaly extended to the pelvis and Vertical supranuclear gaze palsy was detected.

During the recent months, hypotonia after laughter and gelastic cataplexy were observed. Considering neurovascular symptoms, family history, and high NPC risk prediction scores, she was studied for NPC gene mutation and the diagnosis confirmed.

The third case is an 11-months-old boy from the same area, Birjand, South Khorasan, Iran. He is the first offspring of a consanguineous marriage. Due to mild hypochromic, microcytic anemia and splenomegaly were investigated. Liver function tests and enzyme assay for lipid storage disease was normal and foam cells were reported in BMA. He was developmentally age-appropriate. He could crawl and stand with help and speak 1-2 words. Neurological examination of the eye was normal and NPC risk prediction score was 55. He underwent genetic analysis, as well. (Order no: 62266939, Centogene Company) 

In all the three cases, NPC-509 was pathologically increased; they also had an unreported homozygous mutation (c.1415T>C, p.Leu472Pro) in exon 9 of the NPC1 gene.

## Discussion

NPC is a lipid storage disease with autosomal recessive inheritance of incidence rate of 1/120000. In this disease, the age of onset of clinical symptoms and rate of progression of neurological symptoms are highly variable and due to non-specific symptoms, its diagnosis is usually delayed until 5-6 yr after the onset of neurological symptoms (1). The minimizing delays in diagnosis for immediate initiation of miglustat are very important to hinder progression of neurological symptoms and improve treatment outcomes (3, 4). 

The present study aimed to increase awareness and recognition of symptoms for early detection of NPC, especially in Iran, due to the high prevalence of consanguineous marriages.

The true incidence of NPC is higher than the reported rates and these three cases are from Birjand, South Khorasan, with a population of 200000 people. We introduced three cases of NPC, two of which were diagnosed at the age of three yr and one case at one. 

All the three patients were studied due to systemic symptoms. In two cases, diagnosis was made after the neurological symptoms appeared, while for the other one, the disease was diagnosed before the onset of neurological symptoms. Two cases with neurological symptoms were considered in the late infantile (2-6 yr) group. In 31% of NPC patients, the age of onset of neurological symptoms is in the same age range (5).

The first and second cases were cousins and the third case shared the same family of origin. Considering the hereditary nature of NPC, having a sibling is a strong predictor of the disease and having a cousin with NPC is specific, but it is not very sensitive as only 3% of NPC patients had a cousin, involved (6).

Splenomegaly, which is a systemic sign of disease, appeared in all the three patients. In this age group, it might appear at birth or during the first months of life and can be the only clinical finding for many years. 

Isolated unexplained splenomegaly should always be the differential diagnosis for NPC and an indication for investigation of other clinical symptoms and repeated neurological evaluation (2).

Vertical supranuclear gaze palsy (VSGP), the hallmark and strongest indicator of NPC, is seen in 66% of patients. VSGP accompanied with other signs such as splenomegaly and ataxia is highly predictive of NPC, observed in every two patients with neurological symptoms (1). Gelastic cataplexy is characterized by episodes of cataplexy, and is one of the most powerful predictive factors of NPC, observed in one of our patients.

BMA was carried out in each of the three patients due to splenomegaly, demonstrating foam cells.

Moreover, enzyme assay showed normal activity of sphingomyelinase; in NPC, activity of this enzyme is normal despite A and B types. Based on systemic and neurological symptoms and through NPC risk prediction score, If NPC is strongly suspected, mutation of NPC- 1 and NPC-2 genes must be investigated, which is not only important for documentation, but also for prenatal diagnosis and carrier identification (4).

In all the three cases, NPC-509 was pathologically increased; they also had an unreported homozygous mutation (c.1415T>C, p.Leu472Pro) in exon 9 of the NPC1 gene.

It is located in a weakly conserved nucleotide and highly conserved amino acid position, with moderate physicochemical differences between the amino acids leucine and proline. Software analysis by Polyphen -2, SIFT and Mutation Taster predict this variant is probably damaging. To date, this variant is not described in the Exome Aggregation Consortium, Exome Sequencing Project or the 100 Genomes Browser. The current number for identified NPC1 disease-causing mutations is near to 300.

In the NPC1 gene, one mutant allele, p.I1061T, is the most common (approximately 20%-25% of alleles in patients diagnosed in France or the United Kingdom) (1). In the homoallelic state, it correlates with a juvenile neurologic onset form of the disease. 

The second common NPC1 mutation in Europe is p.P1007A. “The mutation p.G992W, typical of Nova- Scotian patients is sporadically found (but rarely) in patients of another origin” (7). For NPC, it has been possible to make genotype- phenotype correlations that predict disease severity and allow more precise genetic counseling (8).

The genotype - phenotype studies in NP-C1 patients showed good correlation between nonsense or frameshift mutations and the most severe neurologic course (7). 

Patients often first present to general practitioners to nonspecific symptoms, the disease often remains undetected or diagnosis is made with many years delay (4).

We should increase awareness about NPC among physicians to improve early diagnosis because early detection of NPC is essential so that therapy with miglustat (Zavesca, Actelion Pharmaceuticals Ltd) that acts as a competitive inhibitor of the enzyme, glucosylceramide synthase. Miglustat can delay onset of neurological symptoms and prolong survival (9). 

**In Conclusion, **we recommend that if NPC is suspected in an infant with systemic signs such as unexplained splenomegaly with or without hepatomegaly, prolonged neonatal cholestatic jaundice, hydrops fetalis and high score in NPC Suspicion Index tool, he/she should be referred to an NPC center for immediate genetic analysis. 

Documentation this mutation is specific to our people and genotype-phenotype correlations for this specific mutations needs to be further studies.
